# Lung Microphysiological System Validates Novel Cell Therapy for Acute Respiratory Distress Syndrome

**DOI:** 10.1002/adbi.202500225

**Published:** 2025-11-20

**Authors:** Bokyong Kim, So‐Hui Kim, Jieun Kim, Eun‐Young Eo, Hyung‐Jun Kim, Jae Ho Lee, Choon‐Taek Lee, Taeho Kong, Su Kyoung Seo, Seunghee Lee, Jeongbin Park, Young‐Jae Cho

**Affiliations:** ^1^ Division of Pulmonary and Critical Care Medicine, Department of Internal Medicine, Seoul National University College of Medicine Seoul National University Bundang Hospital 82, Gumi‐ro 173 Beon‐gil, Bundang‐gu Seongnam‐si Gyeonggi‐do 13620 Republic of Korea; ^2^ School of Biomedical Convergence Engineering Pusan National University Busandaehak‐ro 49 Beon‐gil Yangsan‐si Gyeongsangnam‐do 50612 Republic of Korea; ^3^ Stem Cell and Regenerative Bioengineering Institute Global R&D Center Kangstem Biotech Co., Ltd Ace Highend Tower 8, 84, Gasan Digital 1‐ro Geumcheon‐gu Seoul 08590 Republic of Korea

**Keywords:** acute respiratory distress syndrome, endothelium, mesenchymal stem cells, microphysiological system, translational research

## Abstract

Acute Respiratory Distress Syndrome (ARDS) is a life‐threatening condition characterized by severe inflammation and lung damage, leading to critical hypoxemia. Despite its high mortality rate, the only currently available treatment, Dexamethasone, is associated with significant side effects. This study aims to evaluate the efficacy of primed human umbilical cord blood‐derived mesenchymal stem cells (hUCB‐pMSCs) as a potential alternative treatment for ARDS. A novel lung microphysiological system (MPS) modeling the lung environment is developed and treated with lipopolysaccharide (LPS) to simulate ARDS. The effects of hUCB‐pMSCs and dexamethasone are compared using state‐of‐the‐art methods, including fluorescence‐based imaging and single‐cell RNA sequencing. The hUCB‐pMSCs significantly activated angiogenesis‐related pathways in endothelial cells and enhanced the formation of tip‐like endothelial cells involved in new blood vessel formation. These findings are corroborated by fluorescence microscopy, demonstrating the robust potential of hUCB‐pMSCs as a therapeutic approach. Overall, the results support the potential of hUCB‐pMSCs as a promising alternative treatment for ARDS.

## Graphical abstract

This study aims to evaluate the efficacy of primed mesenchymal stem cells (pMSCs) as an alternative treatment for Acute Respiratory Distress Syndrome (ARDS), based on a novel lung microphysiological system (MPS) to test the efficacy of pMSCs over the standard treatment, Dexamethasone. A pMSCs activate angiogenesis‐related pathways are found in endothelial cells, suggesting the therapeutic potential of pMSCs for ARDS.

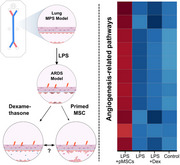

## 1 Introduction

Acute Respiratory Distress Syndrome (ARDS) is a severe respiratory condition marked by rapid‐onset inflammation and pulmonary edema, leading to life‐threatening hypoxemia. Despite its high mortality rate and the scarcity of therapeutic options, effective treatments for ARDS remain elusive. Currently, dexamethasone is the only pharmacological intervention demonstrated to be effective in clinical trials for ARDS.^[^ ^1^ ^]^ However, its use is associated with significant side effects, such as immunosuppression and glucose intolerance.^[^ ^2^ ^]^ This highlights the pressing need for alternative therapies that address the underlying mechanisms of ARDS while minimizing adverse effects.

In recent years, mesenchymal stem cell (MSC)‐based therapies have gained attention as promising candidates for treating ARDS and other inflammatory lung diseases due to their immunomodulatory and regenerative properties.^[^ ^3^, ^4^, ^5^ ^]^ MSCs have been shown to secrete various anti‐inflammatory cytokines, modulate immune responses, and promote tissue repair, making them compelling for addressing lung injury. Preclinical studies in animal models have demonstrated that MSC administration reduces inflammation and facilitates alveolar repair in ARDS. For example, MSC treatment has been shown to mitigate cytokine release and enhance lung function in rodent models of ARDS.^[^ ^6^ ^]^ Furthermore, early‐phase clinical trials have indicated the safety and feasibility of MSC therapy in patients with ARDS, though efficacy data are limited and often inconclusive.^[^ ^7^ ^]^ In addition, recent studies have suggested that primed MSCs are one of the promising therapeutic options in lung inflammatory diseases for their enhanced retention and engraftment efficiency.^[^ ^8^, ^9^, ^10^ ^]^ However, the therapeutic effect of primed MSCs against ARDS remains understudied.

Traditional cell culture methodologies and animal models used in preclinical studies have significant limitations, as they often fail to accurately replicate human lung physiology and the complex cellular interactions within the alveolar environment. To overcome these challenges, we employed a microphysiological system (MPS), commonly named organ‐on‐a‐chip,^[^ ^11^ ^]^ which mimics the dynamic mechanical and biological properties of human lung tissue, providing a more physiologically relevant platform for studying ARDS and evaluating therapeutic interventions.

In this study, we utilized the lung MPS to induce acute lung injury and compared the effects of a novel primed human umbilical cord blood (hUCB)‐derived mesenchymal stem cells (pMSCs) therapy with dexamethasone treatment. The bronchial epithelium represents a significant site of injury during ARDS, where inflammation and tissue damage occur. Accordingly, we constructed our model using human bronchial epithelial cells co‐cultured with endothelial cells. By integrating single‐cell RNA sequencing (scRNA‐seq) to analyze cellular responses at a granular level, we aimed to elucidate the unique mechanisms through which MSC therapy affects inflammation and tissue repair in ARDS.

## 2 Results

### 2.1 Establishment and Visual Assessment of the Lung MPS for pMSCs Treatment Efficacy

**Figure 1:**
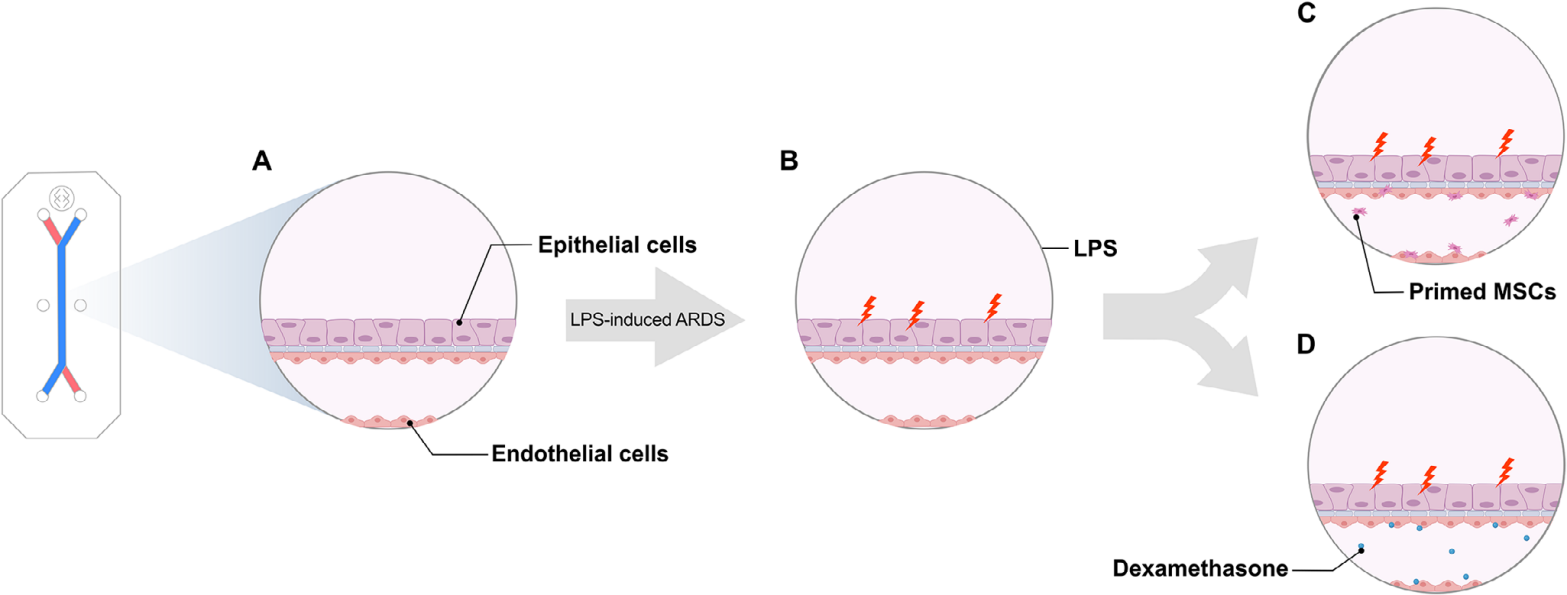
Schematic overview of the LPS‐induced ARDS model and subsequent therapeutic interventions. The figure illustrates the establishment of the acute respiratory distress syndrome (ARDS) model using lipopolysaccharide (LPS) induction, followed by the application of pMSCs and dexamethasone. Key steps include LPS injury to induce ARDS, cell therapy with pMSCs, and dexamethasone treatment used as a positive control for therapeutic efficacy assessment. The diagram includes the following groups: A) Normal lung MPS group; B) LPS‐injury group; C) Cell therapy with pMSCs (primed MSCs) treatment group; D) Dexamethasone (Dex) treatment group.

We established an MPS in vitro using the commercially available Emulate Chip‐S1, creating a microenvironment that mimics normal lung conditions. We modelled ARDS on the lung MPS by injecting LPS. Two medications, dexamethasone and pMSCs, were injected into the ARDS model to investigate the response from each treatment. **Figure** **1** shows a schematic representation of the four control or experimental groups.

**Figure 2:**
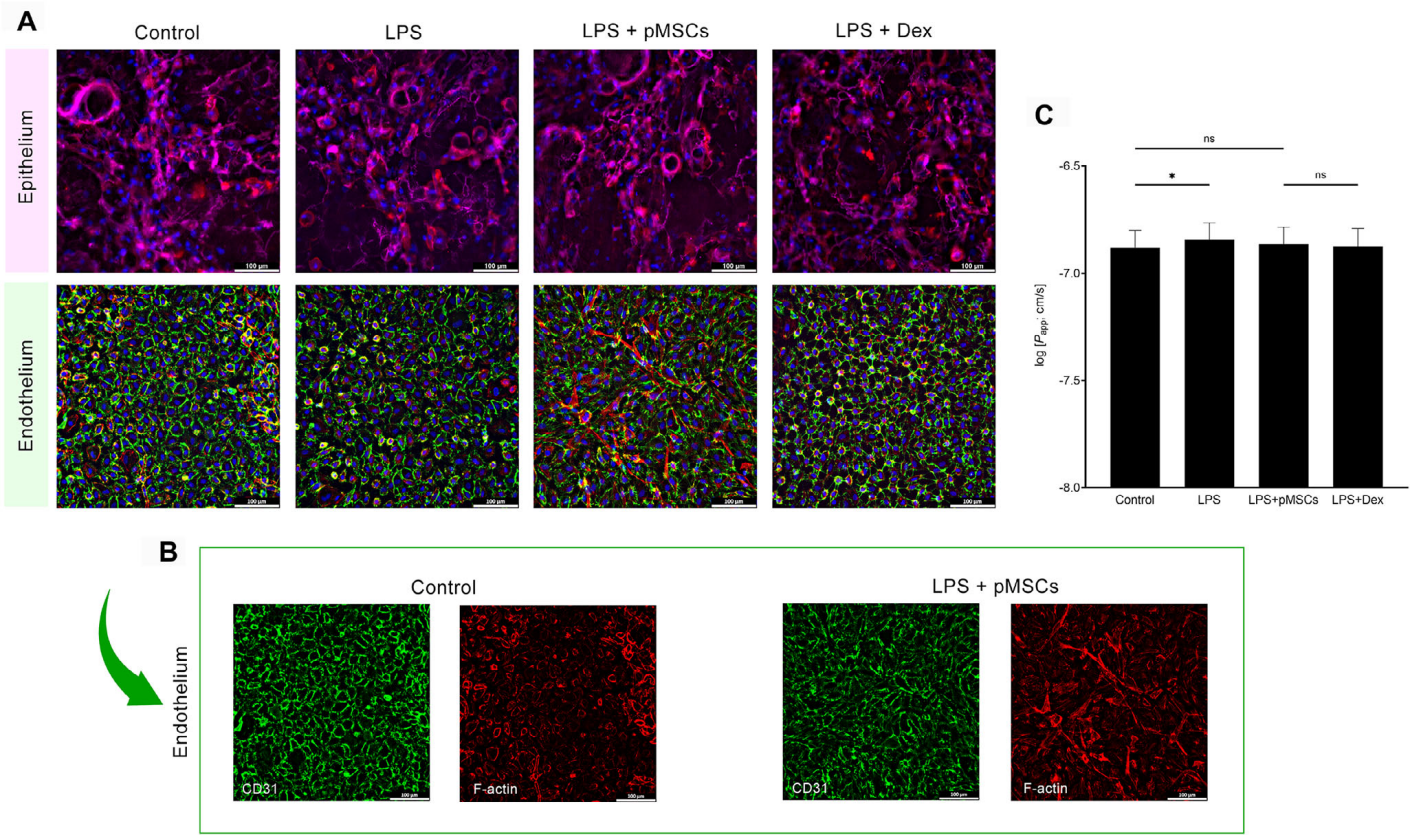
Immunofluorescence and Barrier assessment results from the LPS‐Induced ARDS model on a microfluidic chip. The figure shows A) merged images of epithelial and endothelial cells stained for specific markers: magenta indicates E‐cadherin (epithelial marker), red indicates F‐actin (cytoskeletal marker), green indicates CD31 (endothelial marker), and blue indicates DAPI (nuclear stain). B) Images for the control and primed MSCs‐treated group highlight differences in endothelial cell morphology, with specific staining for CD31 and F‐actin to illustrate the morphological changes induced by primed MSCs treatment. C) Permeability was assessed using Cascade Blue (3 kDa), with differences between groups expressed as mean ± SEM. Statistical significance, determined using one‐way analysis of variance (ANOVA), is denoted as ^*^ *p* < 0.05, ^**^ *p* < 0.01, ^***^ *p* < 0.001, and ns (not significant).

The immunofluorescence analysis results of the control and experimental groups, constructed using the LPS‐induced ARDS model, are illustrated in **Figure** **2**. We examined cell morphology in the top and bottom channels of the lung MPS, employing specific markers: E‐cadherin (epithelial cells), CD31 (vascular endothelial cells), and F‐actin (cytoskeleton). No significant differences were observed between the control and experimental groups in the epithelial cells of the top channel, while slight morphological changes were noted in the vascular endothelial cells of the bottom channel treated with primed MSCs Figure 2B.

To compare morphological changes clearly, images are separated by markers CD31 and F‐actin. The primed MSCs‐treated group exhibited weaker CD31 expression in some regions than the control group; however, compensatory elongated cell shapes were observed in these regions with F‐actin markers. The graph in Figure 2C depicts the evaluation of barrier function through apparent permeability assessment. A significant difference (*p* < 0.05) was observed between the control and LPS‐injury groups; however, no significant difference was noted between the control and primed MSCs‐treated groups. The absence of a significant difference between the control and primed MSC‐treated groups may suggest that the cells were restored to a state comparable to that of the control group.

### 2.2 scRNA Sequencing Analysis

**Figure 3:**
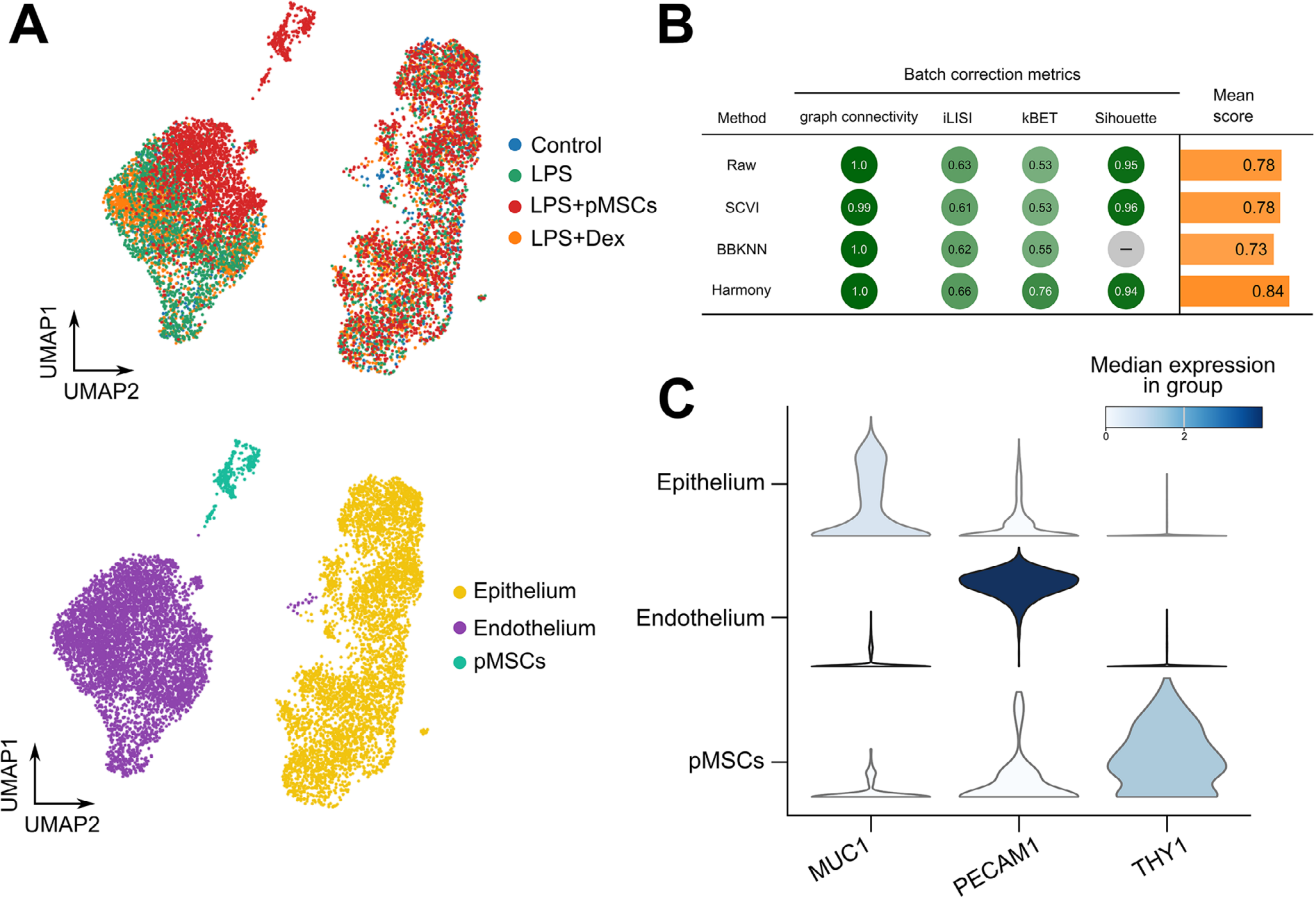
Overview of scRNA‐seq analysis. A) UMAP plot of four samples (left; Control, LPS, LPS+pMSCs, LPS+Dex), and three cell types identified by Leiden clustering (right; Epithelium, Endothelium, and pMSCs). B) Batch correction metrics calculated with epithelial cells, before and after applying three different batch correction algorithms (SCVI, BBKNN, and Harmony). C) Marker gene expression of the three cell types (PECAM1, MUC1, and THY1).

To further analyze the significant results observed in Figure 2, scRNA‐seq analysis was performed using cells collected from the LPS‐induced ARDS model. The clustering of cells resulted in three cell types: epithelium, endothelium, and primed MSCs (**Figure** **3**A).

The identity of the three cell types was validated with the known marker genes (Figure 3C). As Figure 2A shows no significant morphological difference in epithelial cells, the batch correction metrics calculated with epithelial cells were almost as good as the data after applying integration methods (Figure 3B). Thus, we proceeded with the downstream analysis without batch correction.

**Figure 4:**
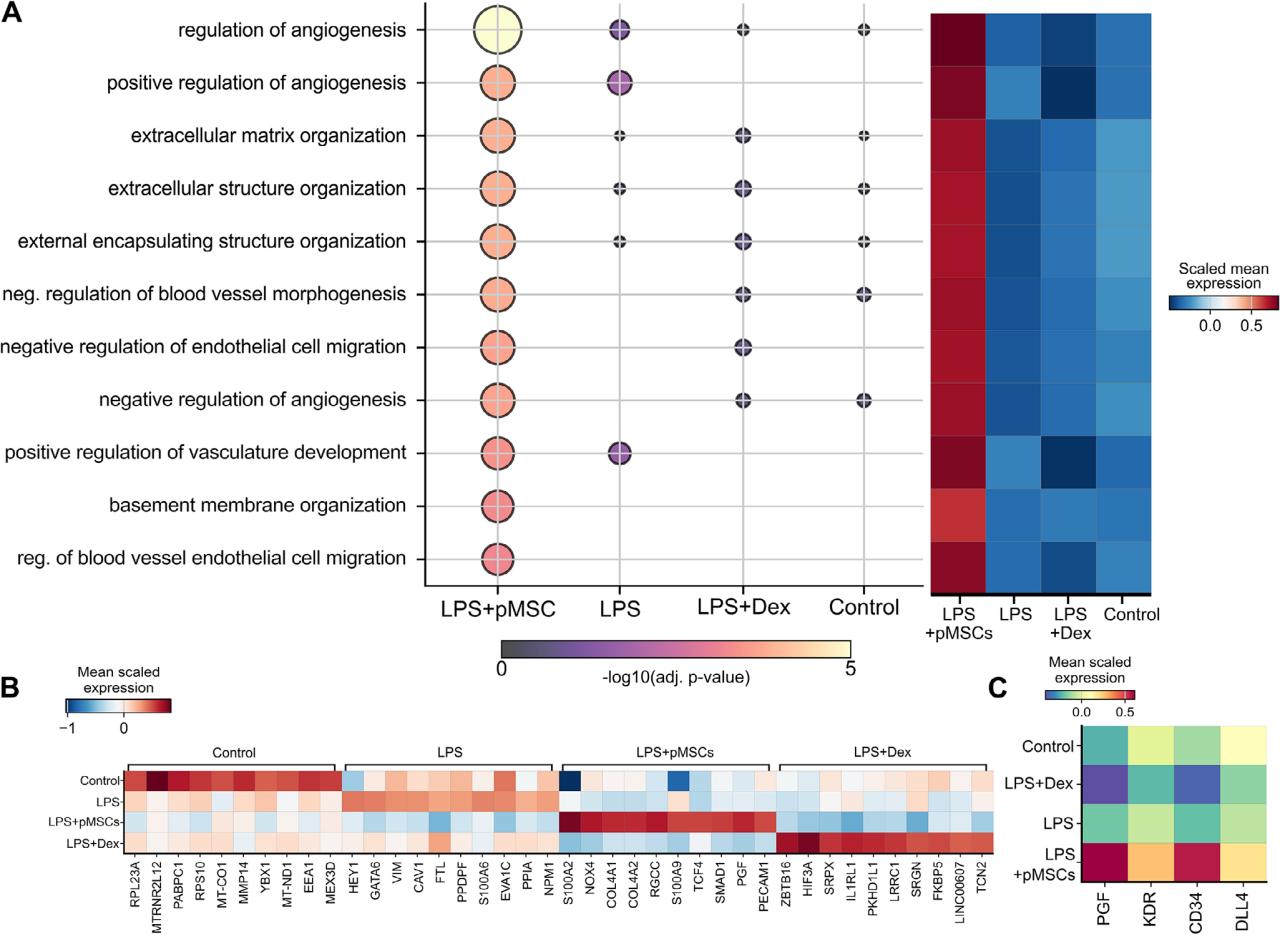
Differential expression analysis reveals elevated angiogenesis pathway in pMSCs‐treated group of endothelial cells. A) Enriched (‐log10(adj. *p*‐value > 3) pathways in the pMSCs‐treated group of endothelial cells and scaled mean gene expression of each pathway. B) Top 10 upregulated differentially expressed genes in each sample, represented as mean scaled gene expression. C) Heatmap of genes related to tip‐like endothelial cells.

Notably, we observed a high cellular heterogeneity within endothelial cells. To characterize the underlying biological features, we conducted differential expression analysis between the four samples using endothelial cells (**Figure** **4**B; to see gene expression heatmap of top 10 genes, see Figure S3, Supporting Information), pathway enrichment analysis filtered by the negative logarithm of adjusted p‐value greater than three, and mean expression of each pathway (Figure 4A). The pathways related to angiogenesis, ECM organization, and endothelial cell migration were highly enriched in the pMSCs‐treated sample compared to the others. Specifically, angiogenesis‐related and extracellular matrix organization‐related pathways were highly associated with the pMSCs‐treated sample (Figure 4A; for other samples, see Figure S1, Supporting Information). Interestingly, we observed the upregulation of the PGF, KDR, CD34, and DLL4 genes, related to tip‐like, angiogenic sprouting in endothelial cells (Figure 4C).^[^ ^12^ ^]^

### 2.3 Observation of Acute Lung Injury Biomarkers and Network Interactions

We established an LPS‐induced ARDS model and conducted a comprehensive analysis to confirm the efficacy of cell therapy by introducing immunomodulatory stem cells to the vascular endothelial channel. This included visual analysis, barrier function assessment, and in‐depth gene‐level analysis through single‐cell RNA sequencing. Subsequently, to verify efficacy at the protein level, we performed cytokine analyses. Cytokine data were obtained from cell supernatants collected on day 3 post‐treatment with primed MSCs and dexamethasone. The cytokine array kit used in this study is primarily optimized for analyzing inflammatory markers. Considering that one of the key pathways of ARDS is the inflammatory response, this study focused on analyzing these inflammatory markers.

The ARDS biomarkers are detailed in **Table** **1**. We categorized each biomarker based on its pathways and referenced the corresponding studies. From the cytokine data, we identified some ARDS biomarkers, as listed in Table 1. The changes in epithelial and endothelial cells were compared between the primed MSCs‐treated and dexamethasone‐treated groups against the LPS‐injury group (Figure S5, Supporting Information). As shown in the heatmap, ARDS biomarker expression exhibited both similar and contrasting patterns between the primed MSCs‐treated and dexamethasone‐treated groups. Biomarkers related to inflammatory pathways, such as IL‐1ß, CXCL8, IL‐10, and TNF‐α, exhibited comparable expression patterns in epithelial and endothelial cells across the two treatment groups. However, some cytokines, such as IL‐6exhibited contrasting expression patterns between the two groups. Notably, among ARDS biomarkers, VEGF exhibited strong expression in the endothelial cells of the pMSCs‐treated group. This finding suggests a potential correlation with the promotion of angiogenesis via the VEGFR‐2 pathway, as indicated in the scRNA‐seq results, which appears to extend to the protein level.

**Table 1:** Acute Lung Injury Biomarkers. This table lists the biomarkers associated with acute lung injury, categorizing by pathway, biomarker, and their respective references from relevant literature.

Pathway	Biomarker	Cited references
Inflammation	IL‐6	Han S, et al. 2015,^[^ ^13^ ^]^ Cabrera‐Rivera GL, et al. 2022,^[^ ^14^ ^]^ Ge et al. 2023,^[^ ^15^ ^]^ Fan W, et al. 2024^[^ ^16^ ^]^
	TNF‐α	Ware LB, et al. 2000,^[^ ^17^ ^]^ Fan W, et al. 2024 ^[^ ^16^ ^]^
	IL‐8	Parsons PE, et al. 2005,^[^ ^18^ ^]^ Spadaro S, et al. 2019,^[^ ^19^ ^]^ Cesta MC, et al. 2021,^[^ ^20^ ^]^ Fan W et al. 2024 ^[^ ^16^ ^]^
	IL‐1ß	Cross LJ et al. 2011,^[^ ^21^ ^]^ Spadaro S et al. 2019,^[^ ^19^ ^]^ Wang Y et al. 2019^[^ ^22^ ^]^
	IL‐18	Spadaro S et al. 2019,^[^ ^19^ ^]^ Mehta P, et al. 2024^[^ ^23^ ^]^
	IL‐10	Cross LJ, et al. 2011,^[^ ^21^ ^]^ Sun Z, et al. 2023^[^ ^24^ ^]^
	IL‐1RA	Cross LJ, et al. 2011,^[^ ^21^ ^]^ Kovach MA, et al. 2015 ^[^ ^25^ ^]^
	sTNF‐RI/II	Martire A, et al. 2016^[^ ^26^ ^]^
Epithelial Injury	SP‐D	Cross LJ, et al. 2011^[^ ^21^ ^]^
	KL‐6	Park M, et al. 2022^[^ ^27^ ^]^
	CC16	Kropski JA, et al. 2009,^[^ ^28^ ^]^ Wutzler S, et al. 2012^[^ ^29^ ^]^
Endothelial Injury	Ang‐2	Cross LJ, et al. 2011,^[^ ^21^ ^]^ Agrawal A, et al. 2013^[^ ^30^ ^]^
	Ang‐1	Spadaro S, et al. 2019,^[^ ^19^ ^]^ Spadaro S, et al. 2021^[^ ^31^ ^]^
	vWF	Spadaro S, et al. 2019,^[^ ^19^ ^]^ Elfawy DM, et al. 2021,^[^ ^32^ ^]^ Qiao X, et al. 2024^[^ ^33^ ^]^
	VEGF	Kong Y, et al. 2020,^[^ ^34^ ^]^ Tomita K, et al. 2020,^[^ ^35^ ^]^ van der Zee P, et al. 2020^[^ ^36^ ^]^
Coagulation	PAI‐1	Capelozzi VL, et al. 2017,^[^ ^37^ ^]^ Spadaro S, et al. 2019^[^ ^19^ ^]^
Cell Damage	HMGB1	Young MD, et al. 2023^[^ ^38^ ^]^
	RAGE	Calfee CS, et al. 2008,^[^ ^39^ ^]^ van der Zee P, et al. 2020,^[^ ^36^ ^]^ Fan W, et al. 2024^[^ ^16^ ^]^Ge et al. 2023^[^ ^15^ ^]^
Acute Phase Reactants	CRP	Ge et al. 2023^[^ ^15^ ^]^

**Figure 5:**
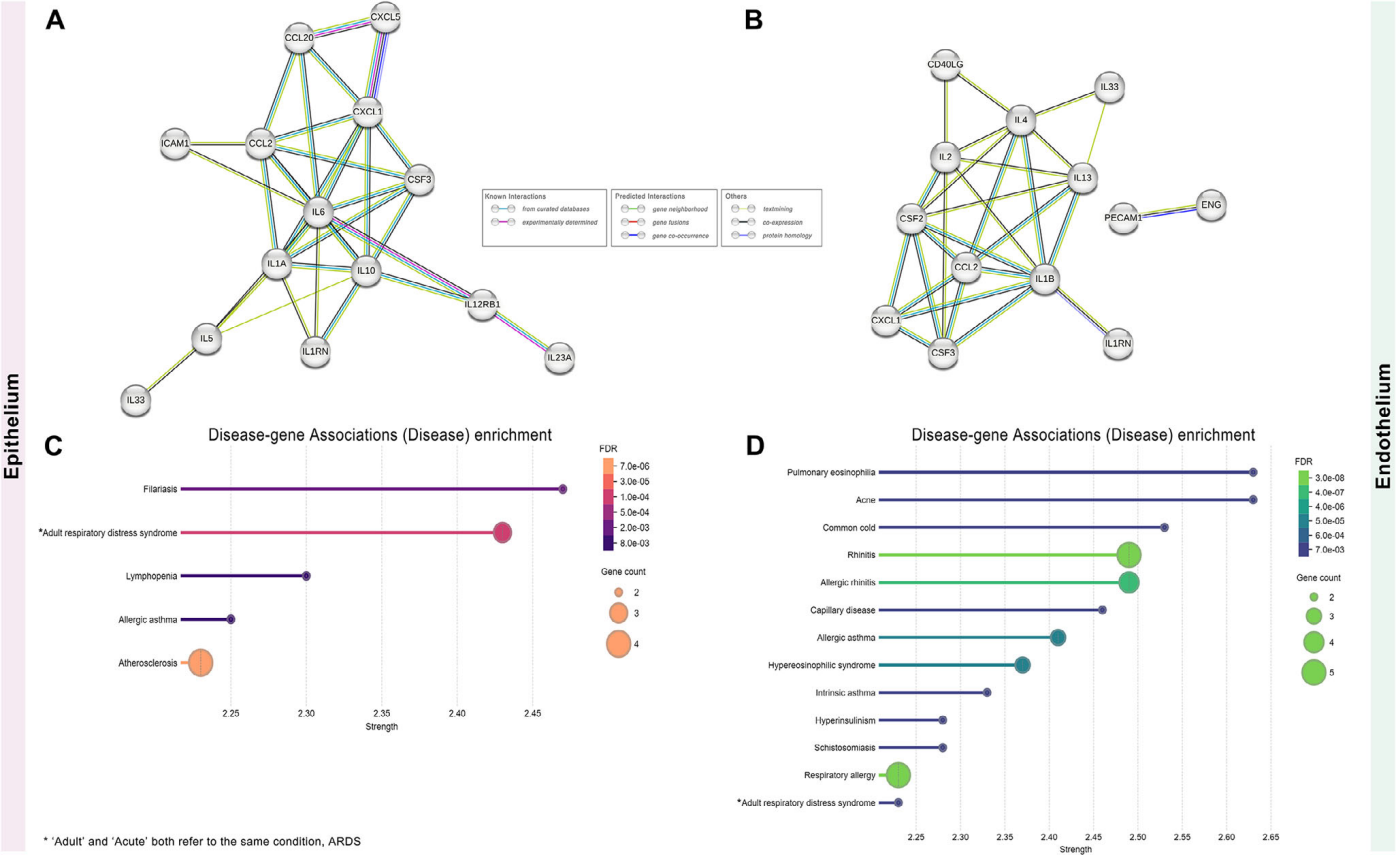
STRING analysis of network interactions for ARDS‐related proteins. This figure illustrates the network interactions identified using STRING analysis based on cytokine array data. The focus is on proteins upregulated in LPS‐injury groups and subsequently downregulated in pMSCs‐treated groups. Analyses were performed with a minimum required interaction score set to the highest confidence (0.9). The figure shows A,B) STRING networks for epithelium and endothelium, highlighting the network interactions for each cell type. Additionally, C) disease‐gene associations for epithelium and D) disease‐gene associations for endothelium are shown, based on the STRING network interactions data. Below each network, functional enrichment visualizations for disease‐gene associations are displayed, sorted by strength, with maximum FDR shown set to FDR ≤ 0.01.

Next, we selected cytokines (or chemokines) that were upregulated in the LPS‐injury group but downregulated in the primed MSCs‐treated group and analyzed the STRING network separately for epithelial and endothelial cells (**Figure** **5**A,B).

The analysis used a minimum required interaction score set to the highest confidence (0.9). Additionally, when visualizing disease‐gene associations based on the STRING network for both epithelial and endothelial cells (Figure 5C,D) we found that epithelial cells had a higher association with ARDS, while endothelial cells had a lower association with ARDS. This suggests that the therapeutic effect was more pronounced in the endothelial cells, consistent with the scRNA‐seq analysis results.

## 3 Discussion

Our study presents an ARDS model developed using biomedical engineering technology, MPS, a platform that has not previously been employed to model ARDS. ARDS leads to damage in both bronchial and alveolar epithelial cells, with inflammation and tissue injury affecting the entire lung. In this regard, the use of NHBEs in our model is particularly relevant, as airway epithelial cells are significantly affected during ARDS and provide critical insights into epithelial damage and repair mechanisms. Therefore, effective treatment should focus on alleviating damage in both cell types. This study is the first to simulate ARDS pathophysiology using the Lung MPS model, a novel platform for ARDS research. Through various efforts, we successfully established a reproducible Lung MPS model with stable cell attachment, utilizing the cells of NHBE and HUVEC. Research with this model provides valuable insights into respiratory epithelial damage and regeneration, contributing to the development of new therapeutic strategies.

The system features a porous membrane that enables direct contact between epithelial and endothelial cells, allowing for a realistic recreation of the lung's 3D architecture in dynamic features generating sheer stress for cells (e.g., blood flow). Also, these capabilities reduce reliance on animal models, prone to interspecies differences and bioethical problems. Based on this, our study demonstrates the successful implementation of an ARDS model and compares the therapeutic efficacy of pMSCs and dexamethasone, the current standard treatment for ARDS.

Interestingly, among the three cell types identified by single‐cell RNA‐sequencing data analysis, the pMSCs‐treated sample differentially expresses genes that stimulate cell proliferation and angiogenic process, including NOX4 and PGF. NOX4 is known as a key regulator of angiogenesis by upregulating the VEGF signaling pathway via VEGFR‐2 phosphorylation and ERK pathway activation, related to endothelial cell migration, proliferation, and angiogenic responses.^[^ ^40^ ^]^ Notably, NOX4 is known to produce hydrogen peroxide that activates NOX2, which is related to elevated mtROS levels, resulting in the enhancement of angiogenesis via upregulated VEGFR2 signaling.^[^ ^41^ ^]^ Furthermore, PGF (placental growth factor) is known to enhance vascular formation by upregulating VEGFR1 (FLT1) and VEGFR2 (KDR) in endothelial cells,^[^ ^42^ ^]^ as validated in mouse models.^[^ ^43^ ^]^ In our data, we found that the gene expression of FLT1 was negligible, but KDR was highly expressed.

Strikingly, previous studies using animal models and the recent one using another MPS for angiogenesis showed that this expression pattern was observed in tip‐like endothelial cells, related to the new formation of vascular vessels at the forefront of vascular sprouts.^[^ ^44^, ^45^ ^]^ These findings can be supported via F‐actin and CD31 fluorescence imaging, where morphological changes of target cells can be linked to the site‐specific attachment of pMSCs (Figure S2, Supporting Information).

Overall, this suggests the considerable potential treatment of ARDS with pMSCs validated through increased gene expression, specifically NOX4 and PGF genes, related to VEGFR‐2 connected angiogenic sprouting, and pMSCs adhesion to the endothelial cells. Additionally, we could not find the genetic markers indicating the predisposition of uncontrolled proliferation of pMSCs, indicating the reliability of the proposed method for ARDS treatment (Figure S4, Supporting Information).

Various priming conditions have been explored to enhance the immunomodulatory capacity of MSCs. In this study, hUCB‐MSCs were primed with IFN‐γ and IL‐1β. It is well established that IFN‐γ induces the secretion of factors such as IDO‐1, IL‐10, PD‐L1, and PGE2, which enhance the ability of MSCs to inhibit T cell proliferation. IL‐1β, on the other hand, is known to promote macrophage M2 polarization.^[^ ^46^, ^47^ ^]^ Additionally, IL‐1β treatment has been shown to stimulate the secretion of VEGF from MSCs.^[^ ^48^ ^]^ Our previous studies demonstrated that pMSCs modulate macrophage M1/M2 polarization and suppress T‐cell proliferation in a mixed lymphocyte reaction (MLR) assay in vitro. In an ARDS mouse model, pMSC treatment resulted in marked anti‐inflammatory effects and increased endothelial cell populations in lung tissue, indicating a potential for vascular regeneration.^[^ ^49^ ^]^ Our findings suggest that the priming process enhances the immunomodulatory and regenerative properties of MSCs, as evidenced by the reduction in inflammation‐related biomarkers in epithelial cells and the increase in angiogenesis‐related biomarkers in endothelial cells. These results highlight the potential of primed MSCs in improving therapeutic outcomes for inflammatory conditions, including ARDS.

Furthermore, diverse evaluation metrics, including permeability assessments and scRNA‐seq analysis, validate the physiological relevance of the ARDS model and underscore the therapeutic promise of pMSCs. Our previous research using pMSCs demonstrated significant anti‐inflammatory effects in an ARDS mouse model and revealed increased endothelial cell populations in lung tissue, suggesting their potential for vascular regeneration.^[^ ^49^ ^]^ These findings are consistent with the present results and support the translational relevance of pMSC‐based therapies.

Our study has several limitations. First, the size of dextran used in this study may not have been sufficient to effectively evaluate vascular or epithelial permeability. As a result, distinguishing differences in the apparent permeability assessment may have been challenging. Future studies will explore the use of dextran of varying sizes to apply more effective verification methods. Second, the lack of sample indexing information required treating cells as samples for differential expression analyses, which may be prone to false discoveries.^[^ ^50^ ^]^ Although we validated that our findings are in high concordance with previous results, additional studies would be beneficial. Third, our study design did not allow for data collection at multiple time points. Future studies will investigate changes beyond angiogenesis by analyzing multiple time points post‐treatment, including both gene and protein levels. Fourth, our in vitro model may not fully recapitulate the in vivo lung environment. To address this, we plan to compare the therapeutic efficacy of primed MSCs in our lung MPS with findings from a mouse model^[^ ^49^ ^]^ and investigate possible side effects and mechanisms of action compared to dexamethasone in vivo. In addition, we acknowledge that ARDS can also be triggered by infectious causes, and our present findings may not fully capture those contexts that need to be addressed in future studies. Lastly, we plan to expand the ARDS model by focusing on alveolar damage and recovery, utilizing human alveolar epithelial and lung microvascular endothelial cells. Through this, we aim to conduct follow‐up studies combining the current Lung MPS model with the expanded model to more comprehensively simulate ARDS.

In summary, our results offer new insights into the potential of MSC‐based treatments for ARDS and highlight the advantages of advanced in vitro models in translational research. These findings, together with results from further studies, are expected to elucidate the therapeutic efficacy and underlying mechanisms of pMSCs. In addition, comprehensive safety analyses will support the development of differentiated cell therapy with enhanced efficacy.

## 4 Experimental Section

### Cell Culture

Primary normal human bronchial epithelial (NHBE) cells (CC‐2540S) and human umbilical vein endothelial cells (HUVEC) (C2517A) were obtained from Lonza (Walkersville, MD, USA). The cells were grown in T75 flasks using BEGM Bronchial Epithelial Cell Growth Medium BulletKit and EGM‐2 Endothelial Cell Growth Medium‐2 BulletKit (Lonza), respectively, at 37 °C, 5% CO_2_, and 95% humidity, with the medium changed every 2 days.

### Integration of Epithelial and Endothelial Cells on Microfluidic Chip

Microfluidic Organ Chips‐S1 (Emulate Inc., Boston, MA) were obtained to ensure the covalent cross‐linking of the ECM polymer to the PDMS. A hydrophilization step was performed to pre‐activate the chip using ER solutions (Emulate Inc., Boston, MA). After activation, both channels were coated with 0.3 mg mL^−1^ collagen type IV (C6745, Sigma–Aldrich) and 0.03 mg mL^−1^ fibronectin (F0895, Sigma–Aldrich), as ECM components. NHBE cells were seeded into the top channel at a density of 3 × 10^6 cells mL^−1^. The epithelial cells were cultured in PneumaCult‐EX PLUS medium (STEMCELL) for 2 days to stabilize cell adhesion. HUVECs were seeded in the bottom channel at a density of 5 × 10^6 cells mL^−1^. The following day, the chips were connected to Pod, portable modules, and placed in a Zoë Culture system. Media for the epithelial and endothelial cells were added to the respective top and bottom channel inlet reservoirs. Microfluidic flow in both channels was set to 30 µL h^−1^ to ensure continuous perfusion. Co‐culture was maintained under continuous flow conditions for an extended period to promote adequate cell‐cell interactions.

### Establishment of an LPS‐Induced ARDS Model

The lung MPS was engineered to replicate the conditions of ARDS. The cells were maintained for 4 days to stabilize the flow rate and to promote cell‐cell interactions through co‐culture. After the stabilization period, lipopolysaccharide from *Escherichia coli* O26:B6 (LPS) (L5543, Sigma–Aldrich) was introduced into the epithelial channel via the top channel reservoir (referred to as the Pod). The LPS was diluted in the epithelial media to a final concentration of 5 µg mL^−1^ and allowed to perfuse through the epithelial channel for 3 days, maintaining flow to target the epithelial cells. The Emulate Chip‐S1 used in the experiment is an MPS with a porous membrane (7‐µm pore) between the top and bottom channels, allowing for material exchange and interaction. The experimental group simulated ARDS conditions by exposing epithelial cells to 5 µg mL^−1^ of LPS for 3 days. The second and third experimental groups also simulated ARDS conditions, each followed by cell therapeutics and drug treatment. In the treated groups, pMSCs or dexamethasone (at a concentration of 140 ng mL^−1^) were infused into the bottom channel, surrounded by vascular endothelial cells, and maintained for 3 days.

### Priming MSCs

hUCB‐MSCs were supplied by GMP Center and cultured in KSB‐3 Basal medium (Kangstem Biotech, South Korea) supplemented with 10% fetal bovine serum (FBS; Gibco) and 100 µg mL^−1^ primocin (Invitrogen). hUCB‐MSCs were primed for 24 h with 20 ng mL^−1^ IFN‐γ (Peprotech) and 5 ng mL^−1^ IL‐1β (Peprotech) to boost their immunomodulatory function or enhance their responsiveness in inflammatory environments. All cells were maintained at 37 °C in a 5% CO2 incubator. Primed human umbilical cord blood‐derived mesenchymal stem cells (pMSCs) were cultured under the same conditions as described in our previous study.^[^ ^49^ ^]^

### Therapeutics Treatment

The pMSCs were thawed and immediately utilized, followed by a series of washes. At the start of the treatments, all reservoirs in each channel were emptied and replenished with the designated cell therapy or drugs. For condition 1, pMSCs were diluted in endothelial cell media (EGM‐2) to achieve a 1:1 cell seeding density ratio with epithelial cells. This mixture was applied to the bottom channel reservoir to ensure exposure to the endothelial channel. Likewise, dexamethasone disodium phosphate (Dex) (Jeil Dexamethasone Inj., Jeil Pharmaceutical Co. Ltd., South Korea) was added to the bottom channel reservoir at 140 ng mL^−1^. Both conditioned chips were flushed at a rate of 600 µL h^−1^ for 5 min to introduce the treatment solutions through each channel, and the original flow rate was restored and applied continuously over 3 days to facilitate sustained drug interaction with the endothelial cells within the bottom channel. Throughout the experiment, a consistent flow was maintained to ensure not only effective treatment delivery but also optimal cellular exposure.

### Immunofluorescence Analysis

Both lung chip channels were fixed with 4% paraformaldehyde in PBS, permeabilized with 0.2% Triton X‐100, and blocked with 1% bovine serum albumin (BSA). The samples were incubated overnight at 4 °C with primary antibodies, E‐cadherin (ab40772, Abcam) and CD31 (ab24590, Abcam), both diluted 1:200 in 1% BSA in PBS. Fluorescence‐conjugated secondary antibodies were applied after dilution in 1% BSA in PBS, at a ratio of 1:200 to 1:500, depending on the efficacy of the primary antibodies. Finally, the chips were stained with a mixture of DAPI (4′,6‐diamidino‐2‐phenylindole) (D1306, Invitrogen) and F‐actin‐555 (R37112, Invitrogen). Imaging was conducted using a Leica DMI8 inverted microscope equipped with the Thunder imaging system.

### Barrier Permeability Assessment

The prepared lung chips were thoroughly inspected for any structural defects before initiating the permeability assay. Based on the permeability assay guidelines provided by Emulate Inc.^[^ ^51^ ^]^ and methods from previous studies using the Emulate S1 chip system.^[^ ^52^, ^53^, ^54^ ^]^ Dextran, Cascade Blue (MW 3 kDa) (D7132, Invitrogen) was diluted to a concentration of 0.1 mg mL^−1^ in PneumaCult‐EX PLUS medium (STEMCELL) and subsequently introduced into the top channel reservoir. The bottom channel reservoir was replenished with fresh EGM‐2 growth media and flushed at a rate of 600 µL h^−1^ for 5 min to ensure that both media reached each channel. A continuous flow rate of 30 µL h^−1^ was maintained for a duration of 2 h. Efflux samples were collected from both the top and bottom channels. The fluorescence intensity of the collected samples was measured using a SpectraMax iD3 Multi‐Mode Microplate Reader (Molecular Devices, LLC., San Jose, CA) at 380 nm excitation and 420 nm emission to quantify permeability across the lung tissue interface. The measured data were then used to calculate permeability values using the Papp Calculator (EC003v1.0, EC004 v1.0) provided by Emulate Inc. The 3 kDa Dextran was employed to detect subtle changes in barrier integrity, as broadly used in epithelial‐endothelial co‐culture microphysiological systems.^[^ ^55^ ^]^

### Single‐Cell RNA Library Preparation

For scRNA‐seq analysis of LPS‐induced ARDS lung MPS, cells were harvested from both microfluidic chip channels and pooled from three chips per group. The pooled cell suspensions were processed for library construction using the Chromium Next GEM Single Cell 3‐Gen Expression v3.1 Dual Index and Chromium Single Cell 3′ Reagent Kit (10X Genomics). Cells were counted and assessed for viability, then diluted to the required concentration of up to 5000 target cells per reaction, ensuring a viability of over 90%. GEMs were formed using the Chromium iX, and libraries were quality‐checked with High Sensitivity D1000 ScreenTape (Agilent) before being pooled for sequencing on the NovaSeq X platform (Illumina) through paired‐end reads with over 20,000 reads per cell.

### Single‐Cell RNA Sequencing Analysis

Raw Fastq files were processed with 10X Genomics CellRanger v7.0.1 with the human reference GRCh38‐2020‐A. The resulting cell‐by‐gene matrices were loaded and concatenated with Scanpy v1.10.3. The low‐quality cells and genes were filtered out. Cell doublets were predicted and removed with Scrublet. The raw counts were normalized and log‐transformed. The top 2000 highly variable genes were selected and used for principal component analysis (PCA), followed by k‐nearest neighbor graph construction. Three clusters were determined using the Leiden algorithm with a resolution parameter of 0.04. The cell types were validated with the known marker genes (PECAM1, MUC1, THY1). Batch correction metrics (graph connectivity, iLISI, harmony, kBET) were calculated with epithelial cells. Among the endothelial cells, the Wilcoxon rank‐sum test was performed between conditions to calculate the differential expression score of each gene. With the top 50 genes having the highest score, the gene enrichment analysis was performed using the “enrichr” function available in GSEApy v1.1.3, based on the GO Biological Process 2021 dataset.

### Cytokine Assay and Data Analysis

The endpoint cell supernatants from LPS‐induced ARDS lung MPS were analyzed for human cytokine and chemokine profiles using the Human XL Cytokine Proteome Profiler Array (ARY022B, R&D Systems, Minneapolis, USA), a membrane‐based antibody array. The intensity of each spot on the array was measured using the Quick Spots Tool (Western Vision Software, Version 22.0.1b), and the average value of the duplicate spots was calculated by analyzing the corresponding pixel densities. A heatmap was created to show expression patterns of ARDS biomarkers in epithelial and endothelial channels, as detailed in Table 1. The heatmap was generated using Seaborn v0.13.2. Cytokine expression data were Z‐transformed to compare ARDS biomarker levels between the primed MSC therapy and dexamethasone treatment groups. Cytokine protein‐protein interaction (PPI) analysis was performed using the STRING database (v11.5, [https://string‐db.org/]). Interactions were identified with a minimum required interaction score of 0.9 (highest confidence), ensuring the inclusion of only the most reliable associations. The resulting network was used to visualize interactions between cytokine proteins and identify key interaction partners.

### Statistical Analysis

Statistical analysis was conducted using GraphPad Prism 9.5.1 to calculate mean and SEM and to perform one‐way ANOVA with Dunn's test (*p* < 0.05). GO analysis was carried out with GSEApy v1.1.3 to identify significant pathways (FDR < 0.001).

## Conflict of Interest

The authors declare no conflict of interest.

## Author Contributions

B.K. and S.‐H.K. contributed equally to this work. B.K., S.‐H.K., J.P., and Y.‐J.C. conceived and designed the work. B.K., S.‐H.K., and J.P. analyzed and processed the obtained data. T.K., S.K.S., and S.L. contributed to the derivation of pMSCs. B.K., E.‐Y.E., and Y.‐J.C. contributed to establishing MPS. Y.‐J.C. and J.P. supervised the work. All authors revised the paper and approved the final manuscript.

## Supporting information



Supporting Information

## Data Availability

The data that support the findings of this study are available from the co‐responding author upon reasonable request.
