# Supplementary material for: Lung Microphysiological System Validates Novel Cell Therapy for Acute Respiratory Distress Syndrome
Source: Adv Biol (Weinh). 2025 Nov 20;10(1):e00225. doi: 10.1002/adbi.202500225 (PMC12789962; doi:10.1002/adbi.202500225)
Supplement: Supplementary file 1 — Supporting Information [file ADBI-10-e00225-s001.pdf]

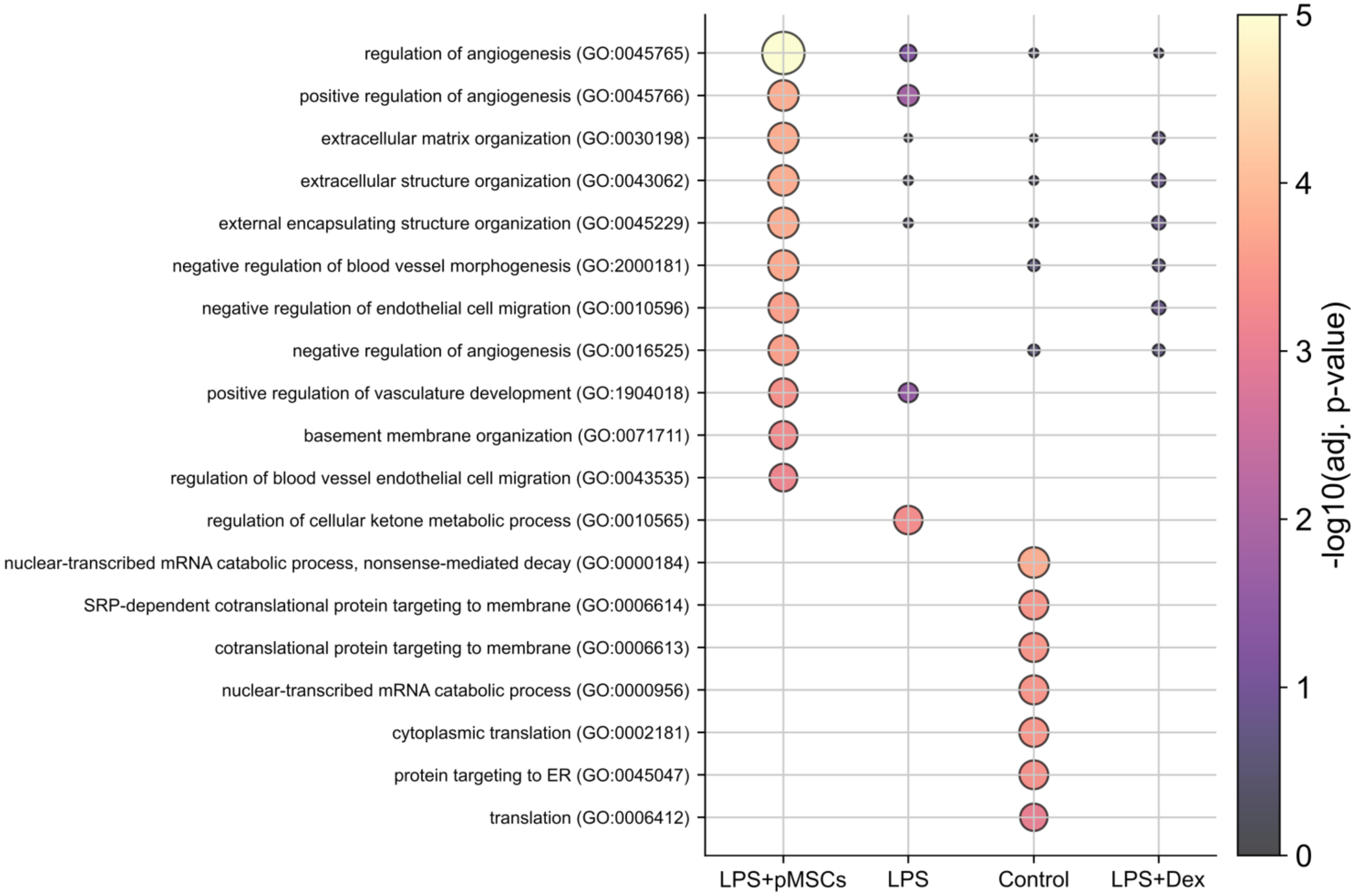

Supplementary Figure 1. Full table of pathway enrichment analysis filtered by the negative logarithm of adjusted *p*-value greater than 3.

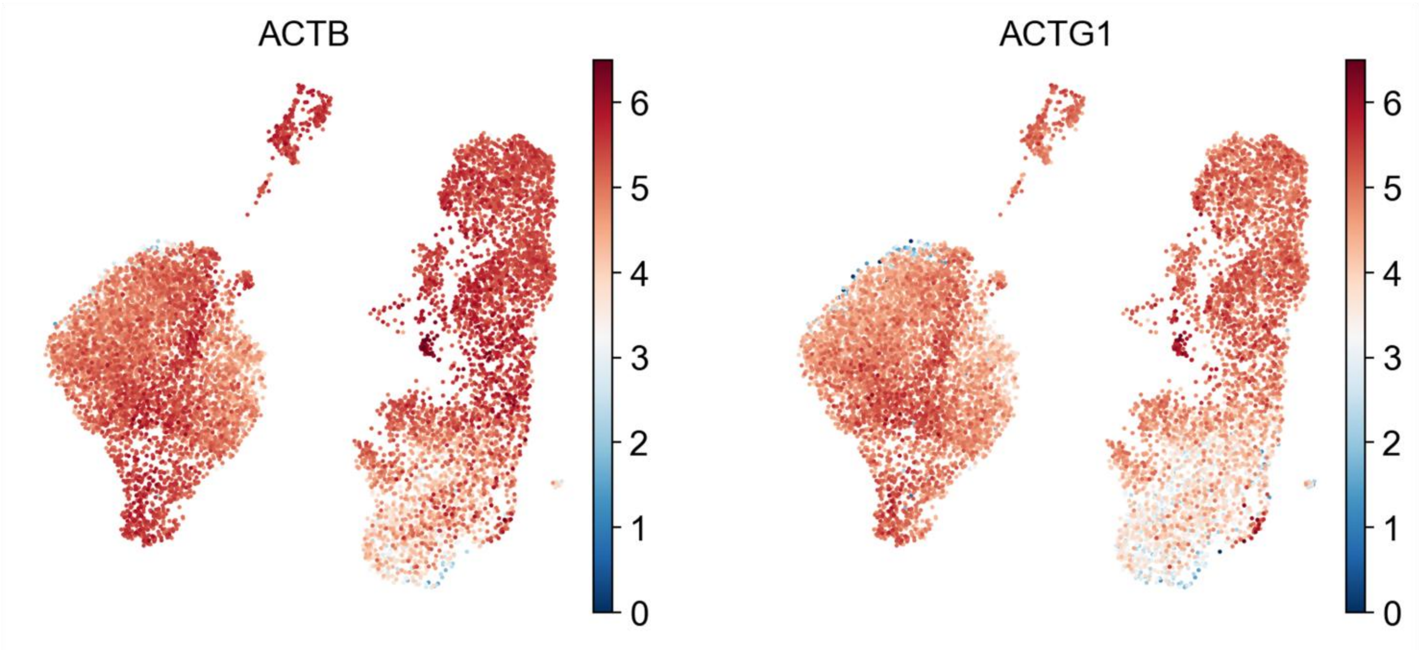

Supplementary Figure 2. UMAP visualization of single-cell RNA-seq data from pMSCs, showing expression levels of F-actin-related genes ACTB (beta-actin) and ACTG1 (gamma-actin). Color intensity represents gene expression levels, with blue indicating low expression and red indicating high expression.

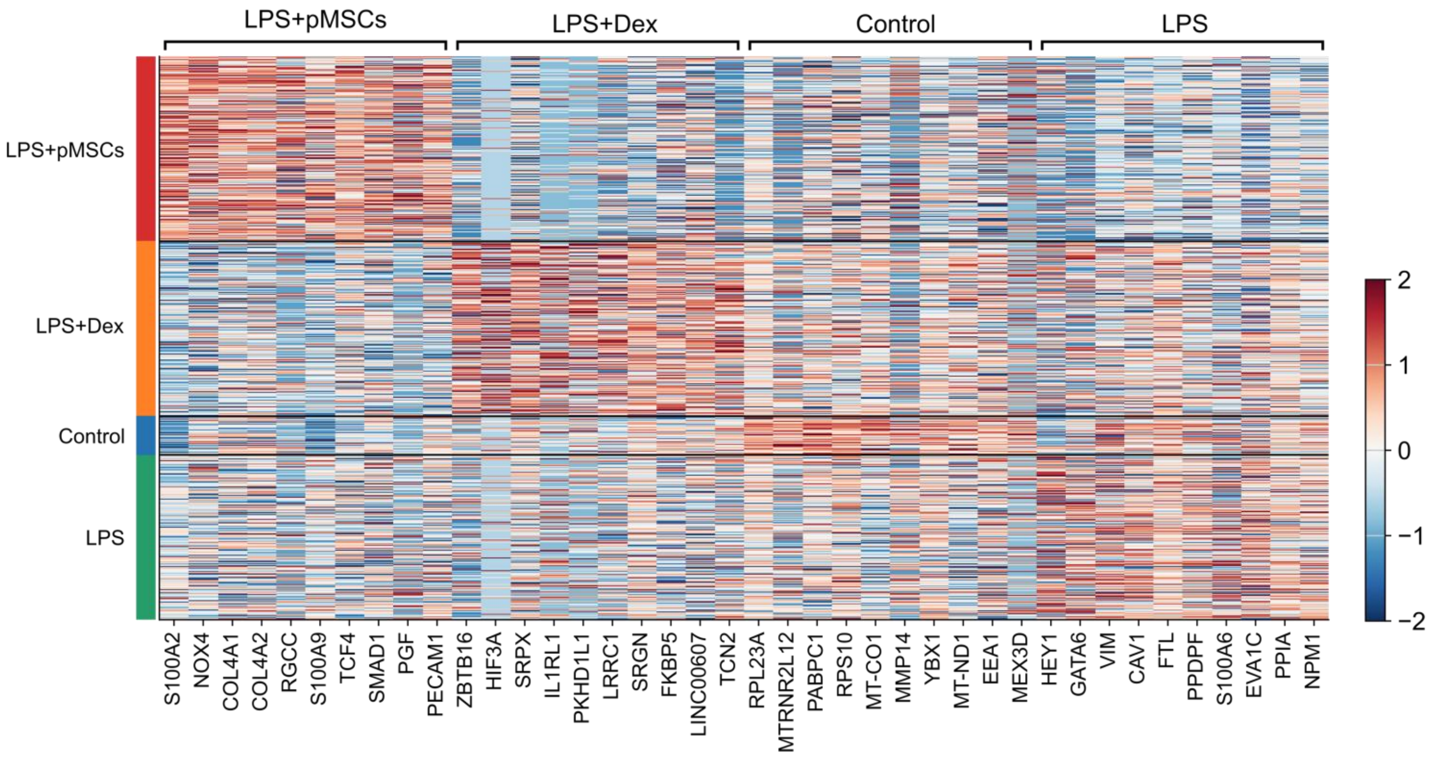

Supplementary Figure 3. The scaled gene expression of top 10 differentially expressed genes from each sample within the endothelial cells.

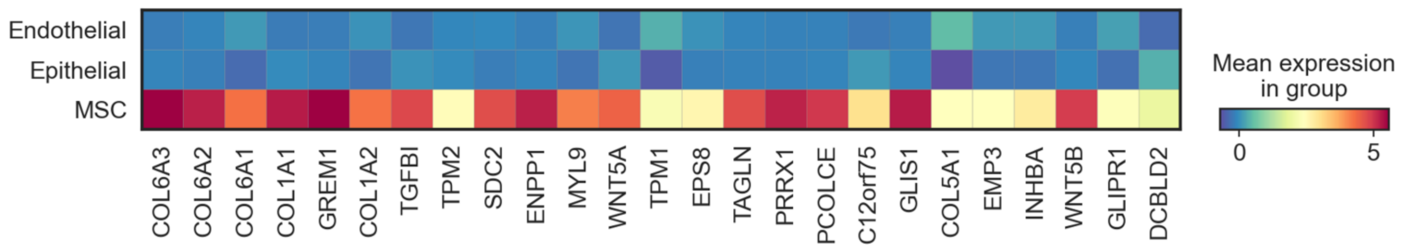

Supplementary Figure 4. Differentially expressed genes in pMSCs represented as mean scaled gene expression.

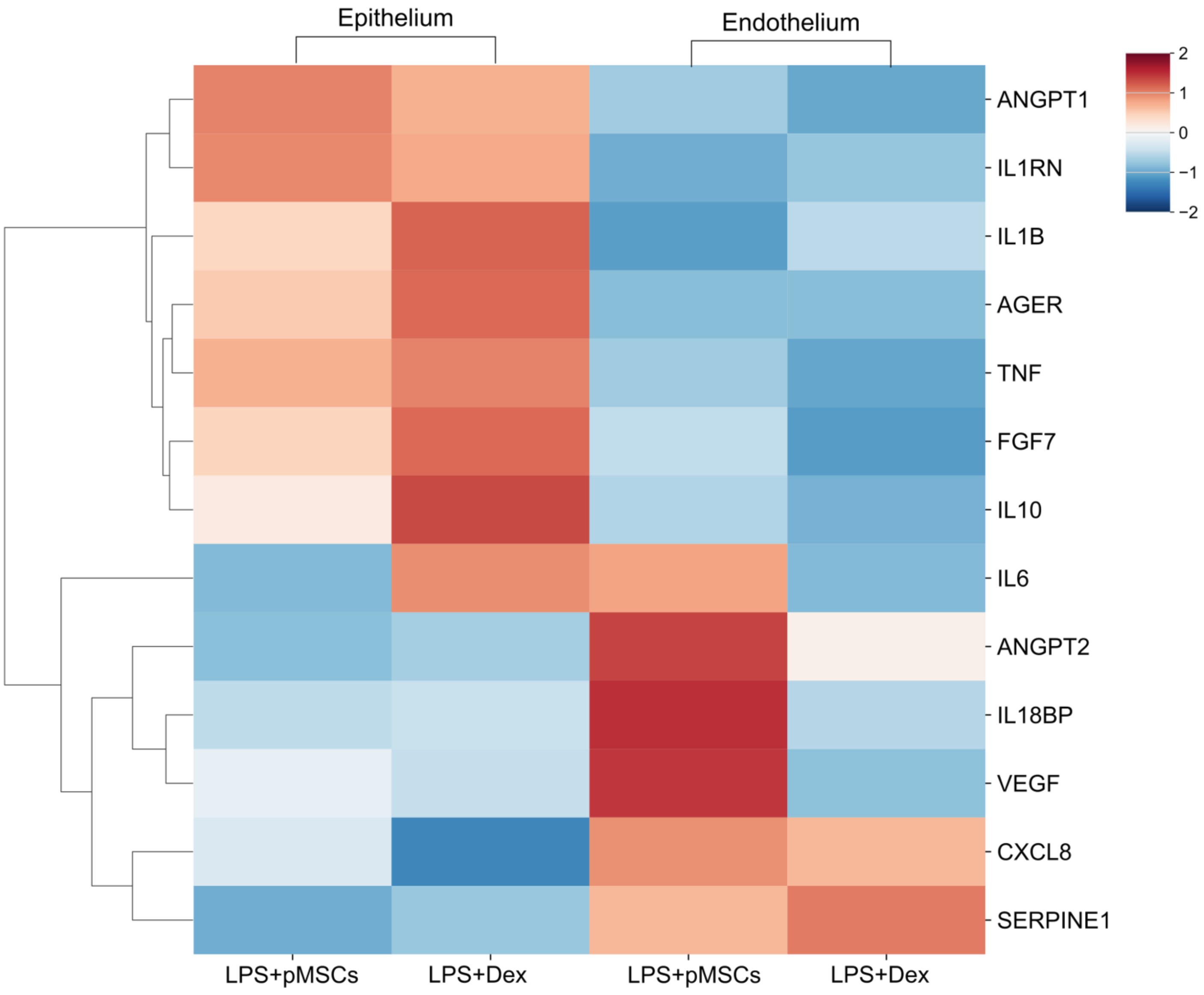

Supplementary Figure 5. Heatmap of ARDS biomarker expression patterns in epithelium and endothelium channels based on semi-quantitative cytokine array results.
